# Predicting response to combination evofosfamide and immunotherapy under hypoxic conditions in murine models of colon cancer

**DOI:** 10.3934/mbe.2023783

**Published:** 2023-09-15

**Authors:** Ernesto A. B. F. Lima, Patrick N. Song, Kirsten Reeves, Benjamin Larimer, Anna G. Sorace, Thomas E. Yankeelov

**Affiliations:** 1 Oden Institute for Computational Engineering and Sciences, The University of Texas at Austin, 201 East 24th St, Austin, TX 78712, USA; 2 Texas Advanced Computing Center, The University of Texas at Austin, 10100 Burnet Rd (R8700), Austin, TX 78758, USA; 3 Department of Radiology, The University of Alabama at Birmingham, 619 19th St S, Birmingham, AL 35294, USA; 4 Graduate Biomedical Sciences, The University of Alabama at Birmingham, 1075 13th St S, Birmingham, AL 35294, USA; 5 O’Neal Comprehensive Cancer Center, The University of Alabama at Birmingham, 1824 6th Ave S, Birmingham, AL 35233, USA; 6 Department of Biomedical Engineering, The University of Alabama at Birmingham, 1075 13th St S, Birmingham, AL 35294, USA; 7 Department of Biomedical Engineering, The University of Texas at Austin, 1107 W. Dean Keeton St, Austin, TX 78712, USA; 8 Department of Diagnostic Medicine, The University of Texas at Austin, 1601 Trinity St Bldg B, Austin, TX 78712, USA; 9 Department of Oncology, The University of Texas at Austin, 1601 Trinity St Bldg B, Austin, TX 78712, USA; 10 Livestrong Cancer Institutes, Dell Medical School, The University of Texas at Austin, 623 W. 38th St Ste 300, Austin, TX 78705, USA; 11 Department of Imaging Physics, The University of Texas MD Anderson Cancer Center, 1400 Pressler St Unit 1472, Houston, TX 77030, USA

**Keywords:** immunotherapy model, mouse, model calibration, uncertainty quantification, tumor growth model, [^18^F]FMISO-PET, hypoxia

## Abstract

The goal of this study is to develop a mathematical model that captures the interaction between evofosfamide, immunotherapy, and the hypoxic landscape of the tumor in the treatment of tumors. Recently, we showed that evofosfamide, a hypoxia-activated prodrug, can synergistically improve treatment outcomes when combined with immunotherapy, while evofosfamide alone showed no effects in an *in vivo* syngeneic model of colorectal cancer. However, the mechanisms behind the interaction between the tumor microenvironment in the context of oxygenation (hypoxic, normoxic), immunotherapy, and tumor cells are not fully understood. To begin to understand this issue, we develop a system of ordinary differential equations to simulate the growth and decline of tumors and their vascularization (oxygenation) in response to treatment with evofosfamide and immunotherapy (6 combinations of scenarios). The model is calibrated to data from *in vivo* experiments on mice implanted with colon adenocarcinoma cells and longitudinally imaged with [^18^F]-fluoromisonidazole ([^18^F]FMISO) positron emission tomography (PET) to quantify hypoxia. The results show that evofosfamide is able to rescue the immune response and sensitize hypoxic tumors to immunotherapy. In the hypoxic scenario, evofosfamide reduces tumor burden by 45.07 ± 2.55%, compared to immunotherapy alone, as measured by tumor volume. The model accurately predicts the temporal evolution of five different treatment scenarios, including control, hypoxic tumors that received immunotherapy, normoxic tumors that received immunotherapy, evofosfamide alone, and hypoxic tumors that received combination immunotherapy and evofosfamide. The average concordance correlation coeffcient (CCC) between predicted and observed tumor volume is 0.86 ± 0.05. Interestingly, the model values to fit those five treatment arms was unable to accurately predict the response of normoxic tumors to combination evofosfamide and immunotherapy (CCC = −0.064 ± 0.003). However, guided by the sensitivity analysis to rank the most influential parameters on the tumor volume, we found that increasing the tumor death rate due to immunotherapy by a factor of 18.6 ± 9.3 increases CCC of 0.981 ± 0.001. To the best of our knowledge, this is the first study to mathematically predict and describe the increased efficacy of immunotherapy following evofosfamide.

## 1. Introduction

The aberrant growth of tumor cells can lead to an oxygen demand that is higher than the existing vasculature can provide [11]. This lack of suffcient oxygenation and nutrients in tumor cells may result in regions of hypoxia (defined as ≤ 10 mm Hg [7]) [27]. When compared to normoxic tumors (i.e., > 10 mm Hg), hypoxic tumors have been linked with a poorer outcome for several anticancer therapies, including radiotherapy, chemotherapy, and immunotherapy [39, 6, 13, 31, 8]. However, it has been shown that resistance to immunotherapy can be alleviated by combining immunotherapy with hypoxia-activated prodrugs and altering the oxygenation within a tumor [31, 12].

Hypoxia-activated prodrugs are activated only under low oxygen tension, resulting in a hypoxiaspecific release of the active drug in areas of low oxygen, such as solid tumors, while sparing normal tissue that has adequate oxygen levels [38]. For example, under hypoxic conditions, evofosfamide (TH-302) is converted into the alkylating agent bromo-isophosphoramide mustard [38]. When combined with radiotherapy and immunotherapy, evofosfamide can improve treatment outcome [31, 12, 38, 1]. Several clinical trials and *in vivo* preclinical experiments have investigated the effects of evofosfamide in combination with a range of treatment types in cancer with favorable results [31, 38, 17, 19, 3, 15]. For example, Laubach et al. designed a phase I/II study for multiple myeloma, to investigate the effects of combination of evofosfamide and dexamethasone where disease was observed to be stabilized in over 80% of the patients [19]. Hegde et al. evaluated the tumor response to a combination of evofosfamide and immune checkpoint inhibitor, ipilimunab [12]. In this study, of 18 patients (with a variety of primary tumors), three achieved partial response and 12 achieved stable disease [12]. Compared to nonresponders, the responders exhibited improved peripheral T-cell proliferation and increased intratumoral T-cell infiltration into hypoxia regions [12]. In preclinical models, evofosfamide was shown to reduce or eliminate hypoxia in prostate cancer and the combination of evofosfamide and immunotherapy cured 80% of mice. Following treatment, mice treated with evofosfamide (alone or in combination with immunotherapy) demonstrated that less than 7% of the tumor area was hypoxic compared to 33% in control mice [15]. These studies indicate that it may be useful to characterize the oxygenation status of a tumor to determine if evofosfamide should be administered in combination with anti-cancer therapy.

There are several methods for noninvasively quantitatively interrogating tumor oxygen status *via* positron emission tomography (PET; [25]), such as [^18^F]FMISO-PET and FAZA-PET. For example, Reeves et al. quantified tumor hypoxia using [^18^F]-fluoromisonidazole ([^18^F]FMISO)-PET before and after immunotherapy [31]. The data was collected in experiments with mice injected with either MC38 (murine colorectal cancer cells) or E0771 (murine breast cancer cells). The authors demonstrated that within five days of starting immunotherapy, nonresponding colorectal and breast tumors exhibited a significant increase in hypoxia. After identifying the nonresponding tumors, the investigators treated them with a combination of immunotherapy and evofosfamide, resulting in significant improvement in tumor volume control and overall survival. This combination of evofosfamide and immunotherapy led to a significant reduction in hypoxia within hypoxic tumors. Further, Kumar et al. [18] have demonstrated that evofosfamide has a synergistic effect when combined with anti-VEGF inhibitors, indicating it’s potential to enhance tumor response to immunotherapy. Given these promising results on identifying hypoxia as a potential imaging biomarker for immunotherapy response and utilizing that information to enhance the efficacy of subsequent treatment with immunotherapy, it is natural to work towards optimizing this process.

Mathematical models have been developed to study the combination of evofosfamide and combretastin in glioblastoma [26], evofosfamide and erlotinib in non-small cell lung cancer [24], and evofosfamide and radiotherapy in chondrosarcoma [10]. For example, Meaney et al. developed a system of reaction diffusion equations to model a glioblastoma treated with evofosfamide and combretastin [26]. They simulated patients receiving just one of the drugs or their combination. The authors also explored scenario where the drugs were released from the vessels or from nanocells. The parameters of the model were taken from previous mathematical models that were calibrated to experimental data, and their results indicate that the nanocells have the potential to increase the delivery and activation of both drugs. Lindsay et al. developed a stochastic mathematical model, parametrized using clinical and experimental data (e.g., experimental growth rates, cell response to evofosfamide, tumor oxygenation measurements, and pharmacokinetic data), and optimized the treatment protocol of evofosfamide and erlotinib [24]. They found that the optimal treatment protocol to reduce the tumor burden is to sequentially alternate a single dose of each drug while minimizing the time between the evofosfamide and erlotinib doses. Hamis et al. developed a hybrid, multiscale cellular automata model to study solid tumors subjected to evofosfamide and radiotherapy [10]. The model was parameterized by published data from an *in vitro* experiment with multicellular tumor spheroids of human chondrosarcoma. Numerical simulations demonstrated that evofosfamide increased the radiotherapy efficacy in hypoxic tumors. Despite these advances on modeling the combination of evofosfamide with other therapies, the combination of evofosfamide and immunotherapy is currently unexplored by mathematical modeling.

In this contribution, motivated by the results obtained in [31], we develop a mathematical model to reproduce the growth of normoxic and hypoxic tumors, as defined by [^18^F]FMISO-PET imaging data, under different treatment regiments of immunotherapy and evofosfamide [31]. The experimental data collected by Reeves et al. is used to calibrate the model. We employed a staggered Bayesian calibration method to isolate the model parameters within each calibration scenario. This approach is designed to systematically explore the main mechanisms of our model that allow it to fit every experimental scenario. The calibration is completed excluding the data from normoxic tumor cells treated with immunotherapy and evofosfamide, which will be used as a validation scenario. Thus, we develop a framework capable of identifying the effects of combining immunotherapy and evofosfamide, and their roles in reproducing the experimental data.

## 2. Methods

### 2.1. Murine model

As details are described elsewhere [31], here we present only the salient details on how we obtained tumor size changes as function of time and treatment in a murine model of colorectal cancer. MC38 murine colorectal cancer cells were obtained from Kerafast (September 2019). These cells are an immunogenic, grade III adenocarcinoma of colorectal cancer and are characterized by microsatellite instability. They were cultured in Dulbecco’s Modified Eagle Medium (DMEM) with 10% fetal bovine serum (FBS), 2 mmol/L L-glutamine, and 1 mmol/L sodium pyruvate in a humidified incubator with 5% CO2 at 37°C. On day 0, 5 × 10^5^ cells were diluted in 40% Matrigel and 60% serum free DMEM and subcutaneously injected into the upper right shoulder of 6- to 12-week-old C57BL/6 mice (Charles River Laboratories). Tumors were grown until they reached a volume of a 100 mm^3^ (7–10 days post inoculation).

**Figure 1.**
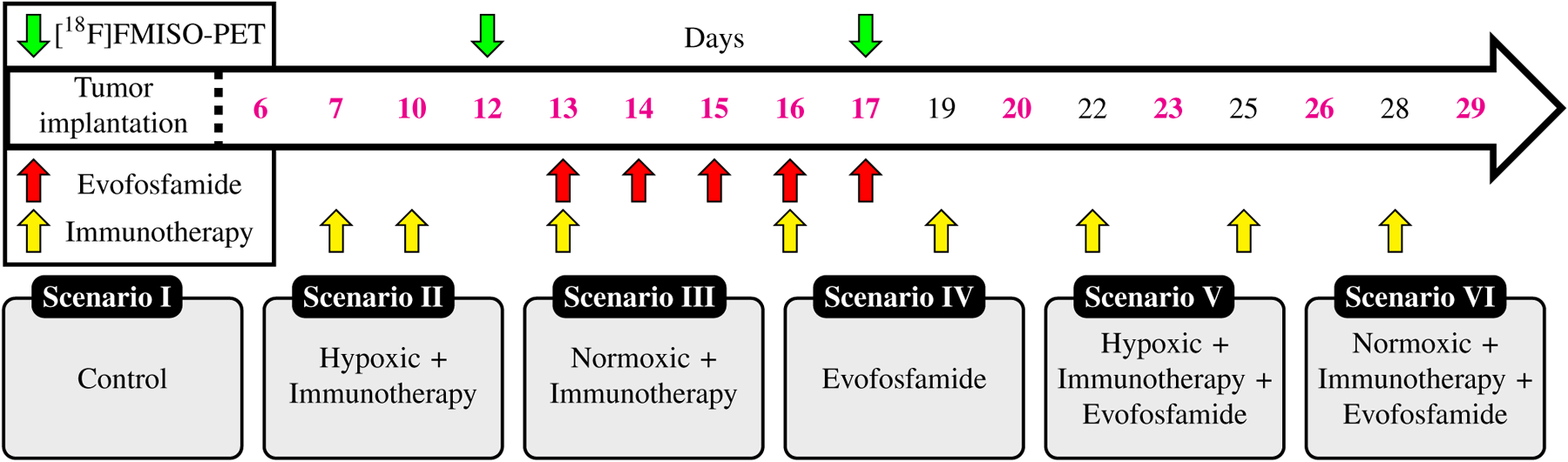
C57BL/6 mice were subcutaneously implanted with MC38 cancer cells. Starting at day 6, the tumor volume was measured with calipers (days marked in magenta). [^18^F]FMISO-PET data was collected at days 12 and 17 (green arrows) to determine percent of the tumor that is hypoxic during treatment. In the days indicated by the yellow arrows, mice received immunotherapy. Mice that received evofosfamide were treated between days 13 and 17 (red arrows). The mice were divided in the six scenarios according to the treatments received and fraction of hypoxic voxels.

In Figure 1, we present the experimental protocol and indicate the days that: 1) [^18^F]FMISO-PET data were collected, 2) mice received 200 *μ*g anti-PD-1 (clone RPM1–14, Bio X Cell) + 100 *μ*g anti-CTLA-4 (clone 9H10, Bio X Cell) combination therapy, 3) mice were treated with 50 mg/kg evofosfamide (TH-302 Selleckchem), and 4) the tumor volume was measured. Immunotherapy and evofosfamide doses were chosen based on concentrations that reflected human clinical studies. To separate mice into normoxic or hypoxic groups, the standard uptake value (SUV) of [^18^F]FMISO in the tumor and muscle were quantified and their ratio was used to stratify tumors as normoxic or hypoxic (SUV>1.86, sensitivity 100%) as previously defined [31]. Thus, mice were divided into six experimental groups (separated based on imaging metrics): I) control, II) hypoxic mice that received immunotherapy, III) normoxic mice that received immunotherapy, IV) evofosfamide, V) hypoxic mice that received immunotherapy and evofosfamide, and VI) normoxic mice that received immunotherapy and evofosfamide.

### 2.2. Mathematical models

We developed a general mathematical model that characterizes the temporal dynamics of the tumor volume by directly accounting for the effects of anti-hypoxia prodrugs, evofosfamide, and checkpoint blockade immunotherapy, anti-PD1 and anti-CTLA4, in different scenarios. We propose a system of ordinary differential equations that captures the temporal evolution of tumor volume, *T*(*t*), and the well-vascularized tumor fraction, *V*(*t*). The well-vascularized fraction is defined as *V*(*t*) = 1 − *H*(*t*), where *H*(*t*) is the hypoxic tumor fraction. We assume that regions with high hypoxia are not vascularized. While hypoxia imaging furnishes valuable insights into a tumor’s oxygenation status [31], our assumption may not fully capture the complexity of vascularization and other factors influencing hypoxia. Nevertheless, given these considerations, we have opted to utilize hypoxia imaging as a proxy for vascularization and have incorporated it into our model. We also assume that the tumor volume increases exponentially at the rate, *k*_*T*_. This proliferation function is selected as the tumor is able to grow without any apparent limiting factor during the time frame from our experiments (e.g., without appearing to reach a maximum tumor volume).

The tumor volume decreases as the immune system eliminates the tumor cells, which we assume occurs more frequently in well-vascularized regions. The tumor has a constant death rate, *μ*_*T*_, but the tumor death rate can increase by *γ*_*i*_ each time, *t*_*i*_, the immunotherapy is delivered. The well-vascularized tumor fraction increases at the rate, *k*_*V*_. This vascularization rate can then increase by a constant,*γ*_*e*_, each time, *t*_*e*_, the mouse receives evofosfamide. As the tumor increases, the hypoxic fraction also increases, and thus, the well-vascularized region decreases at a rate *μ*_*V*_. The pharmacodynamics and pharmacokinetics of the combination of evofosfamide and immunotherapy are defined through *γ*_*i*_, *γ*_*e*_, *r*_*i*_, and *r*_*e*_. The tumor death rate increase, *γ*_*i*_, and vascularization rate increase, *γ*_*e*_, describe the pharmacodynamics of the treatment, as they capture the impact of immunotherapy and evofosfamide on tumor cell death and tumor vascularization, respectively. Conversely, the decay rates of the effects of immunotherapy, *r*_*i*_, and evofosfamide, *r*_*e*_, are vital for describing both the pharmacokinetics of the drugs (and their wash out) in conjunction with the pharmacodynamics with respect to drug efficiency, as they govern the time-dependent decline in the efficacy of both treatments. With these assumptions, the model takes the following form:

(2.1) $$\frac{dT}{dt}=k_{T}T-\left(\mu_{T}+\overset{N_{i}}{\underset{l=1}{\sum }}\gamma_{i}\text{exp}\left(-r_{i}\left(t-t_{il}\right)\right)ℋ\left(t-t_{il}\right)\right)TV,$$

(2.2) $$\frac{dV}{dt}=\left(k_{V}+\overset{N_{e}}{\underset{j=1}{\sum }}\gamma_{e}\text{exp}\left(-r_{e}\left(t-t_{ej}\right)\right)ℋ\left(t-t_{ej}\right)\right)(1-V)-\mu_{V}TV,$$

where *r*_*i*_ and *r*_*e*_ are the decay rates due to checkpoint blockade anti-PD1+anti-CTLA4 immunotherapy and evofosfamide, respectively, and *N*_*i*_ and *N*_*e*_ are the number of days that the mice received immunotherapy and evofosfamide, respectively. In Eqs 2.1 and 2.2, ℋ is the Heaviside function. (See Table 1 for a description of all model parameters.)

### 2.3. Bayesian model calibration

The parameters within Eqs 2.1 and 2.2 are calibrated using the experimental data [31]. To account for the data uncertainties and model inadequacy, the models are calibrated *via* a Bayesian framework defined as:

(2.3) $$\underset{\text{posterior}}{\underset{︸}{\pi (\theta |D)}}=\frac{\overset{\text{likelihood}}{\overset{︷}{\pi (D|\theta )}}\overset{\text{prior}}{\overset{︷}{\pi (\theta )}}}{\underset{\text{evidence}}{\underset{︸}{\pi (D)}}},$$

(2.4) $$\pi (D)=\int_{\Theta }\pi (D|\theta )\pi (\theta )\;\text{d}\theta ,$$

where ***D*** is the experimental data, ***θ*** is the vector of model parameters to be calibrated, ***π***(***θ***) is the prior knowledge about the model parameters (see Table 1 for the prior used for each parameter), ***π***(***D****\****θ***) is the likelihood that the data is observed for a given set of parameters, ***π***(***D***) is a normalizing factor, and ***π***(***θ***|***D***) is the posterior distribution of the parameters. Assuming that the data is normally distributed, the log-likelihood is:

(2.5) $$\ln(\pi (D|\theta ))=\alpha \overset{N_{t}}{\underset{i=1}{\sum }}\left(-\frac{1}{2}\ln\left(2\pi \sigma_{T}^{2}\right)-\frac{{\left(D_{i}^{T}-Y_{i}^{T}(\theta )\right)}^{2}}{2\sigma_{T}^{2}}\right)+\beta \overset{N_{v}}{\underset{j=1}{\sum }}\left(-\frac{1}{2}\ln\left(2\pi \sigma_{V}^{2}\right)-\frac{{\left(D_{j}^{V}-Y_{j}^{V}(\theta )\right)}^{2}}{2\sigma_{V}^{2}}\right),$$

where *N*_*t*_ and *N*_*v*_ are the number of measurements from the experimental data of the tumor and the well-vascularized region, respectively, ***Y*** is the output of the model (i.e., ***Y***^***T***^ is the tumor volume over time, and ***Y***^***V***^ is the well-vascularized fraction over time), and σ_*T*_ and σ_*V*_ are the variance of the total error (i.e., the sum of the variance of the model uncertainties and the model inadequacy) of the tumor and the well-vascularized region, respectively. In our model, we have two quantities of interest, the tumor itself and the well-vascularized region within the tumor. As our goal is to calibrate the model to accurately reproduce the whole tumor volume data, and since we only have two time points to calibrate the well-vascularized region, we assign a higher weight to the tumor (*α*) than to the well-vascularized region (*β*) in the calibration process. Thus, we take *α* = 2 and *β* = 1 for the weights in Eq 2.5.

**Figure 2.**
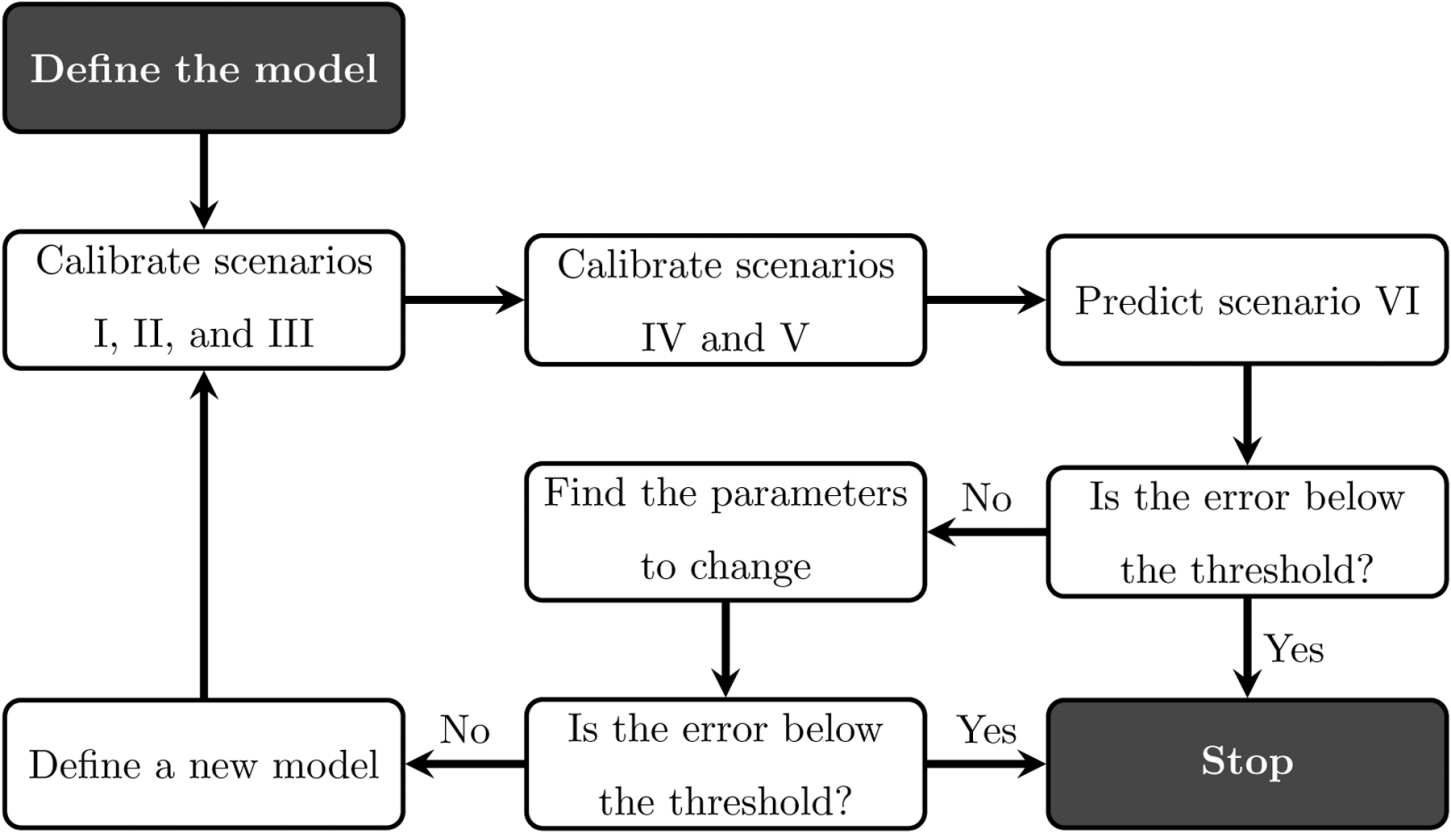
Our model calibration framework begins with first defining an initial model. Next, we calibrate the model parameters to the data from scenarios I, II, and III. The parameters regarding the effects of evofosfamide are excluded from this first set of calibrations, but are determined during calibration to the data from scenarios IV and V. After all the parameters are calibrated, we predict the tumor dynamics in scenario VI and compare the prediction to the data. If the error is below a threshold, we stop the process because we have arrived at a suitable parameter set to describe all scenarios. If the error is above the threshold, we proceed to find which parameter(s) needs to be re-calibrated. If we can reproduce scenario VI with the new parameter value, we then stop. However, if we are not able to capture the tumor dynamics for all scenarios, the mathematical model of Eqs 2.1 and 2.2 would need to be adjusted.

In Figure 2, we present the model calibration framework. We start by defining the complete tumor growth model (i.e., Eqs 2.1 and 2.2), which characterizes the effects of evofosfamide and the immune system. The parameters *k*_*T*_, *k*_*V*_, *μ*_*V*_, *μ*_*T*_, *γ*_*i*_, *r*_*i*_, and the initial well-vascularized fractions are calibrated to scenarios I, II, and III (note that each scenario has its own initial well-vascularized fraction). Thus, we can calibrate just the effects of the immunotherapy and the tumor growth without the interaction with evofosfamide. Next, we calibrate the parameters *γ*_*e*_ and *r*_*e*_, and the initial well-vascularized fractions, to scenarios IV and V while using the values obtained from the previous scenarios to assign the other parameters. After this step, all model parameters are calibrated and we can test the ability of the model to predict the tumor volume over time in scenario VI. If the prediction error (as quantified by, for example, the mean percent error, the Pearson correlation coefficient (PCC), or the concordance correlation coefficient (CCC)) is below a defined threshold, we can conclude that our model is able to accurately predict scenario VI with the parameter values obtained from scenarios I - V. This would mean that only knowledge of the evofosfamide effects on hypoxic cells is sufficient to accurately inform the temporal changes in normoxic tumor cells treated with immunotherapy and evofosfamide combination. However, if we find that the model fails to accurately predict the tumor growth (i.e, the prediction error is above the threshold), we find the smallest number of parameters to re-calibrate to capture the tumor growth observed in scenario VI. If we are able to find this set of parameters, it means that there are further differences between normoxic and hypoxic tumors that are not captured by the model when using the same parameter values obtained from scenario I - V. If we are not able to identify a parameter set to capture the tumor growth in scenario VI, the underlying mathematical model described by Eqs 2.1 and 2.2 need to be changed.

### 2.4. Sensitivity analysis

In this work, we utilize the Sobol method, which is a variance-based global sensitivity analysis method [35, 34, 33]. One notable aspect of this method is its ability to assess the contributions of individual parameters, as well as capture higher-order effects arising from parameter interactions. We now summarize the application of the Sobol method to our problem.

Let $\vec{M}(\vec{\theta })$ be a model parameterized by parameters $\vec{\theta }$ which belong to a parameter space $\vec{\Theta }\subset {\mathbb{R}}^{k}$. Among all possible methods for computing the total sensitivity index (which quantifies all effects of the parameter on the model output), we have selected the techniques presented in [36] due to their efficient convergence with smaller sample sizes compared to other approaches. Initially, we construct two *N* × *K* matrices, **A** and **B**, where *K* is the number of parameters, and *N* is the sample size. In these matrices, *N* random samples are drawn from a uniform distribution corresponding to the range of each parameter’s uncertainty. Each row of these matrices represents a sampled value for $\vec{\theta }$. Additionally, *K* matrices $A_{B}^{\left(k\right)},k=1,2,\cdots ,K,$, are defined where all columns are from **A** except the *k*^*th*^ column, which comes from **B**. The model is then evaluated for each row of the matrices **A** and $A_{B}^{\left(k\right)}$ and the outputs are stored in the vectors ***Y***_***A***_ and $Y_{AB}^{\left(k\right)}$, respectively. Finally, post-processing these results consists of computing the total sensitivity index, $S_{T_{k}}$, for each parameter *k* [14]. The total effect, $S_{T_{k}}$, measures the contribution of the input *θ*_*k*_ to the model output variation. The total-effect index for each parameter, ${\left\{S_{T_{k}}\right\}}_{k=1}^{K}$, can be approximated using the following estimator [36],

(2.6) $$S_{T_{k}}\approx \frac{1}{2N}\overset{N}{\underset{j=1}{\sum }}{\left({\left(Y_{A}\right)}_{j}-{\left(Y_{AB}^{\left(k\right)}\right)}_{j}\right)}^{2}.$$

By employing the algorithm outlined in the previous paragraph, we can estimate multi-dimensional integrals with just *N*(*K* + 1) model evaluations [36]. For time-dependent processes, we repeat these steps for each time step to capture the importance of each model parameter over time. In the results section, we present the time evolution of $S_{T_{k}}$ for multiple treatment protocols, including control, immunotherapy, evofosfamide, and immunotherapy plus evofosfamide. The order of parameter importance can be used to define the order in which to change parameters, as shown in the “find the parameters to change” step presented in Figure 2. Specifically, we re-calibrate the parameter that has the highest ranking and keep the other parameters fixed. This process helps to identify the minimum number of parameters that need to be re-calibrated to accurately capture scenario VI.

### 2.5. Numerical implementation

The model given by Eqs 2.1 and 2.2 and the calibration framework (presented in Figure 2) are implemented in C++. The model is solved *via* a fourth order Runge-Kutta method [4]. We employ a parallel, adaptive, multilevel Markov Chain Monte Carlo (MCMC) sampling method to compute the posterior density *π*(**θ|*D***) (see, e.g., [29, 32, 9]). The MCMC method is available in the C++ library QUESO (Quantification of Uncertainty for Estimation, Simulation, and Optimization) [30]. The code itself, as a well as a description of how to use it, is provided at https://github.com/Ernesto-Lima/CEIRPrediction.

## 3. Results

**Figure 3.**
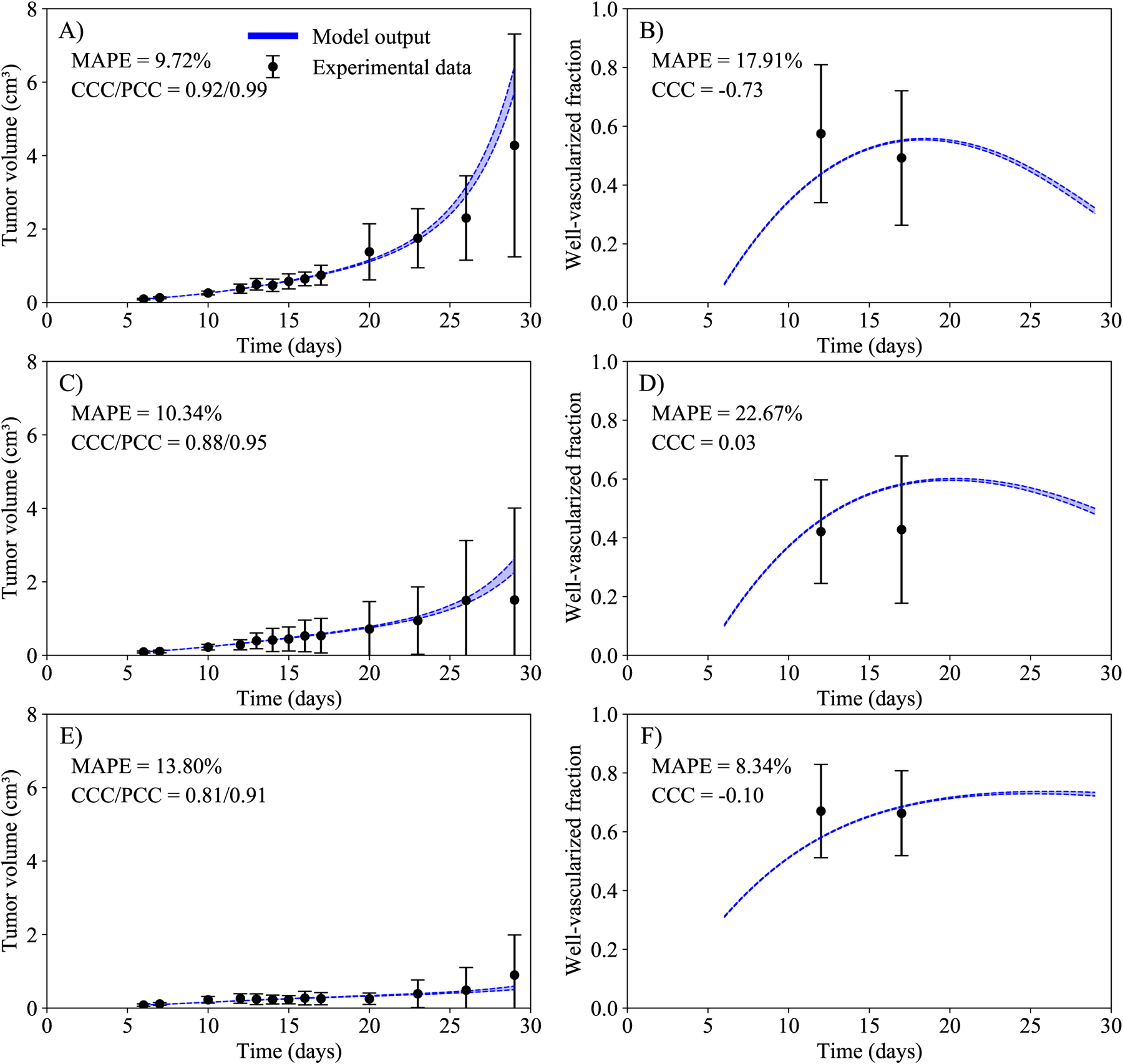
Temporal dynamics of the experimental data (black) and the model (blue) in scenarios I (control, panels A and B), II (hypoxic tumors that received immunotherapy, panels C and D), and III (normoxic tumors that received immunotherapy, panels E and F). Left column represents the tumor volume, and right column the well-vascularized fraction. The dashed lines in blue indicate the bounds of the 95% confidence interval of the model output. The mean and 95% confidence interval from the data is also indicated in black. The mean absolute percent error for the tumor volume is below 14% and the CCC is above 0.8 in the three scenarios calibrated. Even though the calibration is weighted towards the tumor volume, the mean absolute percent error for the well-vascularized fraction is below 23% in the three scenarios.

Using the priors defined in Table 1 and following the framework presented in Figure 2, we calibrate the parameters *k*_*T*_, *k*_*V*_, *μ*_*V*_, *μ*_*T*_, _*i*_, *r*_*i*_, and the initial well-vascularized fractions to the data from scenarios I (control), II (hypoxic tumors that received immunotherapy), and III (normoxic tumors that received immunotherapy). In Figure 3, the experimental data and the calibrated model are presented for these scenarios. The mean absolute percent error for the tumor volume is below 14% and the CCC is above 0.8 in all three calibrated scenarios. Although the calibration is weighted towards the tumor volume, the mean absolute percent error for the well-vascularized fraction is below 23% in all three scenarios. It is worth noting that, while the initial well-vascularized fraction is similar in scenarios I and II (panels B and D, respectively), the initial vascularization is higher in scenario III (panel F), which is the normoxic scenario.

**Figure 4.**
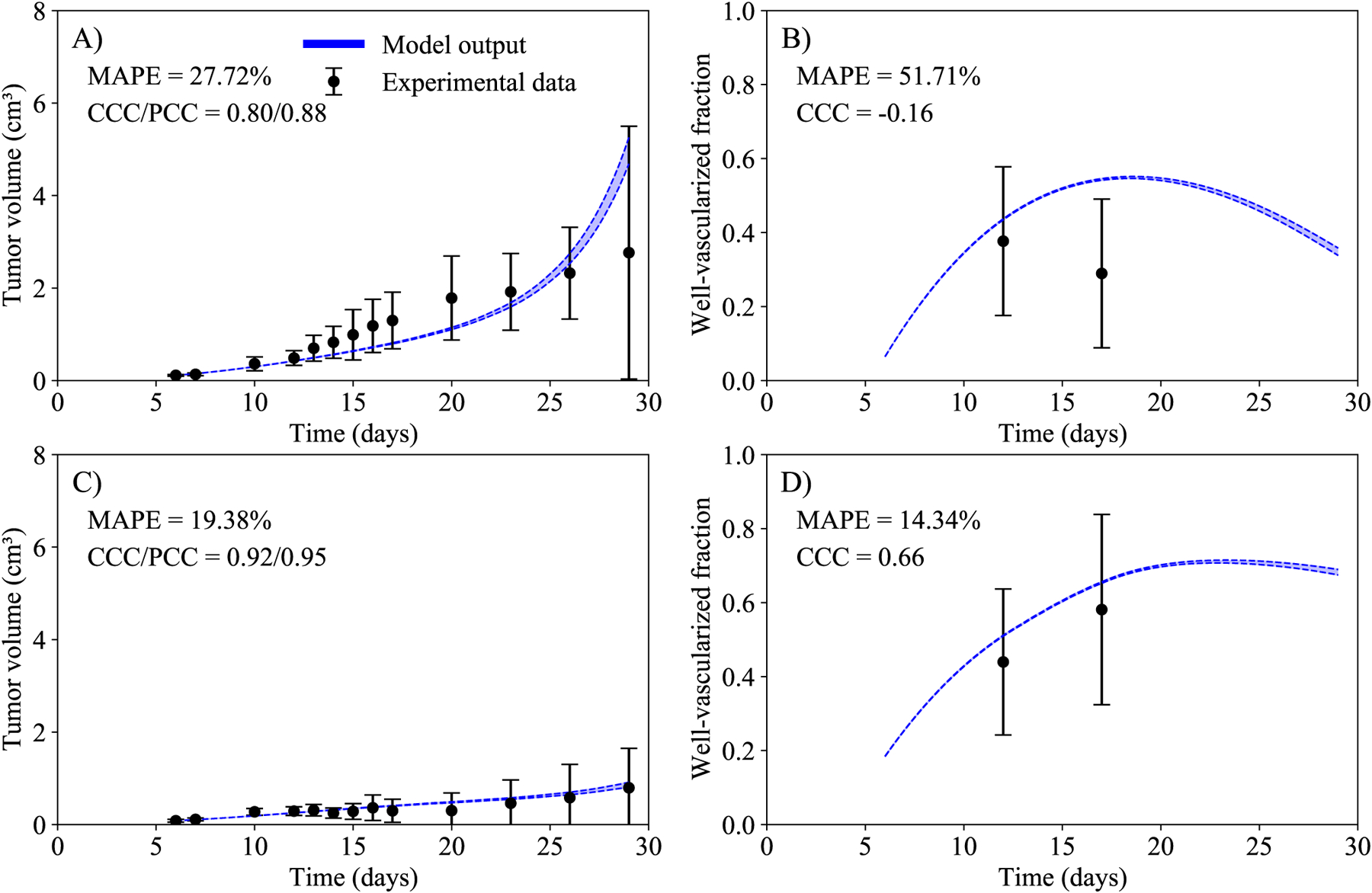
Temporal dynamics of the experimental data (black) and the model (blue) in scenarios IV (evofosfamide alone, panels A and B), and V (hypoxic tumors that received combination immunotherapy and evofosfamide, panels C and D). Left column represents the tumor volume, and right column the well-vascularized fraction. The dashed lines in blue indicate the bounds of the 95% confidence interval of the model output. The mean and 95% confidence interval from the data is also indicated in black. The mean absolute percent error for the tumor volume is below 28% and the CCC is above 0.8 in the two scenarios calibrated.

In the next step of our framework, we calibrate the parameters *γ*_*e*_ and *r*_*e*_, and the initial well-vascularized fractions, to the data from scenarios IV (evofosfamide alone) and V (hypoxic tumors that received combination immunotherapy and evofosfamide). The other six parameters are fixed to the values found during the calibration of scenarios I, II, and III. At this step of our framework, we are able to calibrate every parameter from our model (i.e., Eqs 2.1 and 2.2). In Table 2, we present the mean and the 16th and 84th percentiles (i.e., the standard deviation of a normal distribution) of the calibrated parameters. In Figure 4, the experimental data and the calibrated model are presented for scenarios IV (evofosfamide) and V (hypoxic mice that received immunotherapy and evofosfamide). The mean absolute percent error for the tumor volume is below 28% and the CCC is above 0.8 in the two scenarios calibrated. These results indicated that we are able to capture the tumor dynamics of these two scenarios using the parameter values obtained in scenarios I, II, and III.

**Figure 5.**
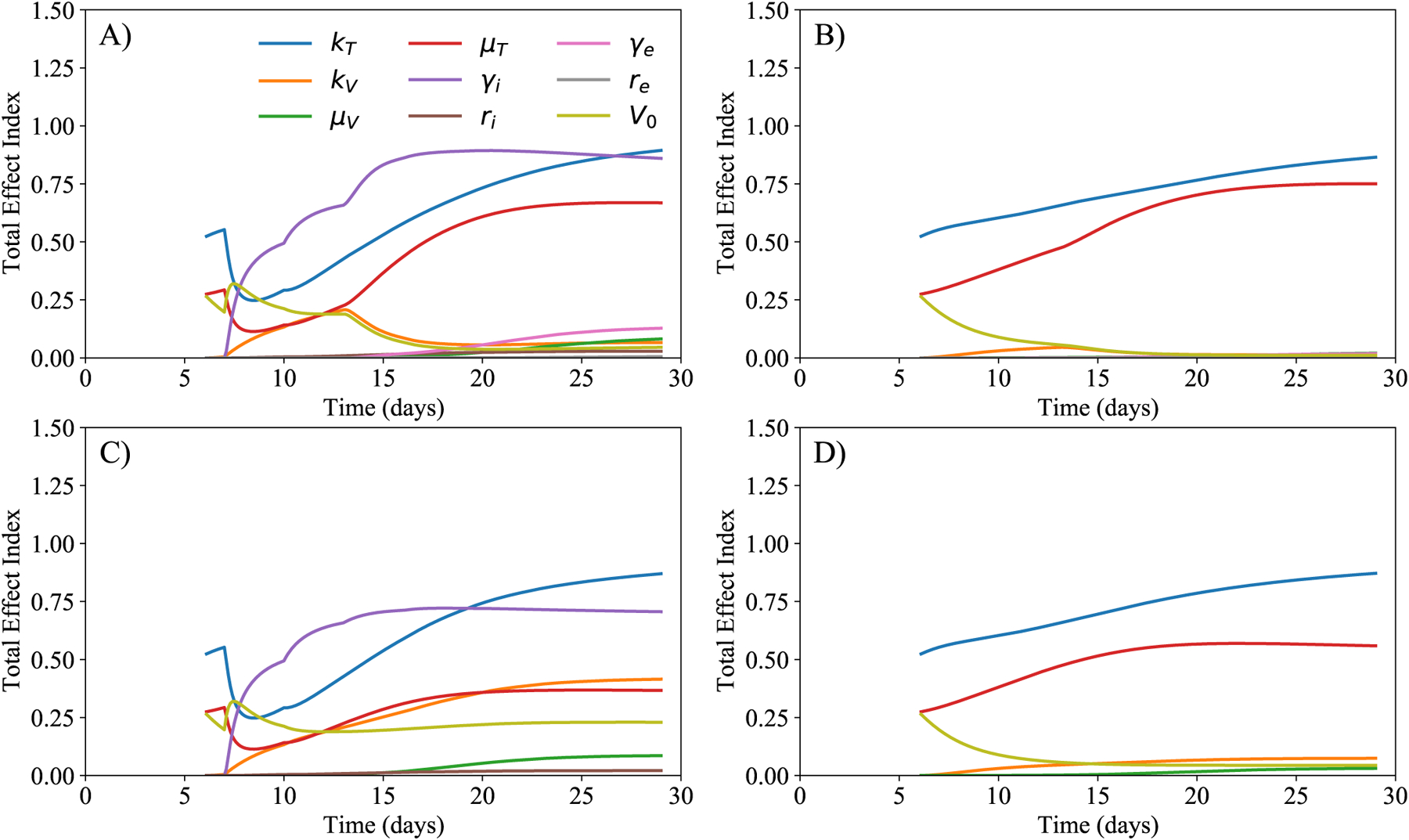
Sensitivity analysis of the model in the following scenarios: immunotherapy plus evofosfamide (panel A), evofosfamide (panel B), immunotherapy (panel C), and control (panel D). The sensitivity index is computed throughout the entire experimental timeline, offering insights into how different factors play a role at different stages of tumor growth and response to treatment. In panels B and D, the tumor growth rate has the highest total effect index (*k*_*T*_, blue line), followed by the death rate caused by the immune system (*μ*_*T*_, red line). However, with the immunotherapy, in panels A and C, the increase in tumor death rate due to immunotherapy (*γ*_*i*_, purple line) has the highest total effect index. This is due to the increase of immune cells when delivering immunotherapy in panels A and C.

**Figure 6.**
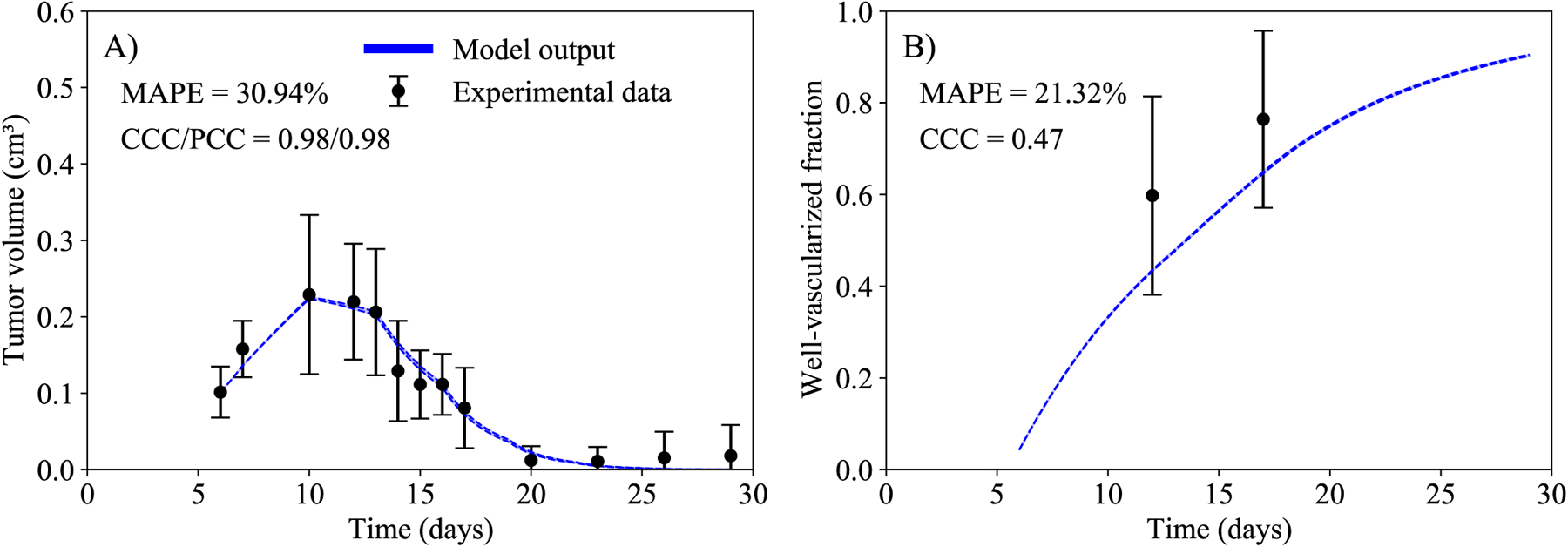
Temporal dynamics of the experimental data (black) and the model (blue) in scenario VI (normoxic tumors that received combination immunotherapy and evofosfamide). Left column represents the tumor volume, and right column the well-vascularized fraction. The dashed lines in blue indicate the bounds of the 95% confidence interval of the model output. The mean and 95% confidence interval from the data is also indicated in black. The mean absolute percent error for the tumor volume is below 31% and the CCC and PCC are both above 0.98.

The last step from our framework is to capture the tumor temporal evolution of the data from scenario VI (normoxic tumors that received combination immunotherapy and evofosfamide). Using the parameter values calibrated to the data from scenarios I-V, our model was unable to accurately predict the tumor dynamics in scenario VI, achieving a CCC of only −0.08). To increase the prediction accuracy (i.e., increase the CCC) of the change in tumor size over time in scenario VI, we fixed seven parameters to the values obtained in calibration steps I - V and calibrated the remaining one to the data from scenario VI. We test each parameter from the highest total-effect index to the lowest. In Figure 5, we present the sensitivity analysis of the eight model parameters and the initial condition of the well-vascularized fraction (*V*_0_) for four scenarios (i.e., immunotherapy plus evofosfamide (panel A), evofosfamide (panel B), immunotherapy (panel C), and control (panel D)). We computed the sensitivity index of each parameter for all time points. Thus, our results cover the same time range as the duration of the experimental data. The sensitivity analysis shows that, without the immunotherapy (i.e., panels B and D), the tumor growth rate is the most important parameter, followed by the death rate induced by the immune system. However, when immunotherapy is delivered (i.e., panels A and C), the increase in tumor death rate due to immunotherapy becomes the most important parameter, followed by the tumor growth rate, and the death rate due to the immune system. It is worth noting that the period during which the increase in tumor death rate due to immunotherapy, *γ*_*i*_, is of greatest importance is prolonged when evofosfamide is administered. In panel A (i.e., immunotherapy plus evofosfamide), the interval of highest importance is between days 7.6 and 26.6 (19.0 days) in the simulation interval of [6,39], while in panel C (i.e., immunotherapy) the interval is between days 7.6 and 19.2 (only 11.6 days, nearly 40% less). Based on these results, we found that we only need to change the value of *γ*_*i*_ to capture the observed changes in tumors size in scenario VI. (Table 2 presented all calibrated values from all scenarios). Note that, to capture the observed tumor volume changes in scenario VI, we need to increase the value of γ_*i*_ by a factor of 18.6 ± 9.3 compared to the value used in the other scenarios. This indicates that, in normoxic tumors, the delivery of evofosfamide dramatically increases the efficacy of the immunotherapy. Interestingly, this increase in *γ*_*i*_ does not translate into a significant change in the total tumor death rate. The total tumor death rate is the tumor death rate by the immune system, *μ*_*T*_, plus the increase in death rate by the immunotherapy, *γ*_*i*_. Thus, the total tumor death rate increases by a factor of 2.52 ± 0.53 (see supplemental Figure S1) when adding evofosfamide into the normoxic tumors treated with immunotherapy. In Figure 6, the experimental data and the calibrated model are presented for scenario VI. The mean absolute percent error for the tumor volume is 30.94% and the CCC is 0.98. The high percent error is due to tumor volumes that reach zero after day 23.

## 4. Discussion

We have developed a system of ordinary differential equations capable of reproducing the tumor and its vascularization fraction growth and decline under the combined effects of immunotherapy and evofosfamide treatment. To the best of our knowledge, this represents the first effort to mathematically describe the interactions of these two drugs. Our model was able to recapitulate the experimental data showing that evofosfamide can rescue the immune response and sensitize hypoxic tumors to immunotherapy [31]. Following the framework presented in Figure 2, we were able to isolate the effects of immunotherapy and evofosfamide. We calibrated the parameters that model the tumor growth and immunotherapy using the data from scenarios I, II, and III (i.e., scenarios in which evofosfamide was not delivered). In scenario II, the hypoxic tumors did not respond to immunotherapy. The next step was to calibrate the evofosfamide terms using the data from scenarios IV and V, while keeping the previously calibrated parameters fixed. In scenario V, the hypoxic tumors responded to immunotherapy following the evofosfamide treatment. Thus, the response of hypoxic tumors to immunotherapy, observed in scenario V, is only due to the terms of our model effected by evofosfamide. More specifically, the increase in tumor vascularization leads to an increase in immunotherapy efficacy. Reeves et al. have shown that evofosfamide can increase tumor vascularization [31], and this may facilitate the delivery of immunotherapy to the tumor cells. However, when predicting the normoxic tumor response to immunotherapy and evofosfamide (i.e., scenario VI), our model was unable to obtain a good agreement with the experimental data. Our model was only able to reproduce scenario VI when the death rate of tumor cells due to immunotherapy increases directly as a result of evofosfamide, rather than solely through an indirect effect due to the increase in tumor vascularization. This finding conflicts with our experimental data, which shows that evofosfamide alone has no effect on tumor size (i.e., scenario IV). However, it should be noted that previous studies have demonstrated that evofosfamide can improve the effectiveness of immunotherapy, in addition to increasing tumor vascularization [16, 12]. Phase I experiments conducted by [16] and [12] have shown that evofosfamide can resensitize tumors to immunotherapy, supporting the idea that it may have a direct effect on the death rate of tumor cells due to immunotherapy. Therefore, our model’s results are consistent with other studies demonstrating that evofosfamide can enhance the effectiveness of immunotherapy. In our results, even though the death rate by immunotherapy, *γ*_*i*_, increased by a factor of 18.6 ± 9.3 from the hypoxic tumor to the normoxic tumor, the total death rate (i.e., the tumor constant death rate, *μ*_*T*_, plus death rate by immunotherapy) increased by a factor of 2.52 ± 0.53, which is consistent with experimental data.

Sensitivity analysis is a valuable technique to help understand the complex behavior of biological systems. To select which parameters would be kept fixed with the values from previous scenarios, and which ones would be calibrated to the data from scenario VI, we tested the parameters according to their sensitivity analysis rank (from most to least influential). By measuring the effects of model parameters on system outputs, sensitivity analysis helps determine the underlying biological mechanisms that govern system behavior [35, 34, 33]. In time dependent biological processes, sensitivity analysis allows for assessing the relative importance of each model parameter over time. For example, in tumor growth models, sensitivity analysis can identify the most critical model parameters that influence the growth and temporal dynamics of cancer [22, 28, 21]. In our study, the results of the sensitivity analysis identify *γ*_*i*_, death rate due to immunotherapy, as the key parameter to change to accurately reproduce the tumor growth dynamics in scenario VI, revealing that in normoxic tumors, there is heightened response to immunotherapy.

There are a few opportunities for further investigation that arise from this work. From an experimental perspective, our analysis is based on data from mice implanted with colon adenocarcinoma cells and may not necessarily be generalizable to other types of tumors. A possible extension would be experiments with different cell lines, and the inclusion of anti-angiogenic therapies (as done in [18]), to verify the synergy between evofosfamide and VEGF inhibitors. Another possible extension is the inclusion of different treatment protocols, altering the order, timing, and sequencing of the therapies. The current results are based on data from a limited number of scenarios and may not accurately capture the full range of possible responses to treatment. Future studies should test how switching the order and doses of the two treatments affect the temporal change in tumor size and hypoxic status. With a broader dataset that included more diverse treatment protocols, the model could be calibrated to predict optimal drug dosing and timing for maximizing the synergistic effects of evofosfamide and immunotherapy (as done in [23]). This predictive capability could be valuable in guiding treatment decisions and designing more effective therapeutic strategies. Additionally, it would be desirable to have measurements on vascularity as afforded by (for example) dynamic contrast enhanced magnetic resonance imaging [41]. While the imaging allowed for tumor information on hypoxia, additional [^18^F]FMISO-PET time points would have provided the opportunity to better characterize the longitudinal changes in hypoxia. Both of these measures would allow for the separation of the vascular and hypoxia contributions to treatment efficacy. This additional imaging data would come with a significant increase in resources and exposure of ionizing radiation to the animals. However, by applying the methods of optimal experimental design (e.g., [20, 5, 2]) to our mathematical model, we can determine the optimal time points for acquiring additional [^18^F]FMISO-PET scans to minimize the uncertainty of our models and increase model’s prediction accuracy. This approach could help us maximize the information obtained from the additional imaging data while minimizing the associated costs. The additional imaging data could help to validate whether evofosfamide can maintain a reduction of hypoxia that can allow for long-term sustained responses to immunotherapy.

From a mathematical and computational perspective, the model is based on a set of ordinary differential equations, which may not fully capture the complexity of the interactions between evofosfamide, immunotherapy, and tumor cells. Investigating spatial heterogeneity and interactions through partial differential equations or agent-based models could improve the model’s accuracy, predictive power, and generate new hypotheses on the underlying biology. Mechanistic models like the quantitative systems pharmacology platform developed by [40] incorporate a more extensive set of parameters (282 parameters) to offer detailed insights into underlying biological mechanisms and interactions. Such models enhance comprehension of the immune-oncology system. However, our platform has an advantage of using a minimal parameter set. This enables model calibration using limited data from a single patient, leading to personalized calibration and reduced uncertainty in predictions. Given the available vascularity measurements, we developed a simplified vascularization model that could still capture the observed phenomena to the best extent possible. While more sophisticated models might be constructed with larger datasets, our model adequately captures the phenomena observed in these experiments. It assumes an increase in vascularization after each evofosfamide treatment, effectively depicting tumor growth across different experimental scenarios. Notably, our model excludes the direct cytotoxic effects of evofosfamide. This assumption aligns with the specific experimental data used to calibrate the model [31]. The experiments indicated minimal direct cytotoxic impact on the tumor volume, although it did influence combination therapy at the cellular level. Consequently, our focus lay in developing a simple model calibrated to the available experimental data, capable of capturing the key observed phenomena. We opted to exclude the direct cytotoxic effects and concentrate on evofosfamide’s role as an oxygenating and sensitizing agent, as evident in the experimental data. However, even in the absence of a term representing the direct cytotoxic effects, evofosfamide indirectly impacts tumors. It increases the well-vascularized fraction, increasing the tumor death rate *via* the immune system’s action through the term *μ*_*T*_*TV*. Finally, the current model is based on the assumption that evofosfamide increases tumor vascularization, facilitating the subsequent delivery of immunotherapy to tumor cells. However, as seen in scenario VI, this is not enough to capture the effects on normoxic cells, necessitating an increase in the death rate due to immunotherapy. Further study is needed to determine the mechanisms behind this effect and how it may be incorporated into the model. Our current model could be adapted and calibrated with experimental data for other therapies that affect oxygenation (or vasculature) to predict their potential synergistic effects with immunotherapy. While the specific effects may vary between different drugs, the efficacy of any hypoxia-activated prodrug in reducing hypoxic regions and enhancing the effects of immunotherapy would be critical for its success.

The results from our model, in combination with the experiments presented in [31], demonstrate that it would be feasible to use of [^18^F]FMISO-PET in conjunction with hypoxia-activated prodrug therapy in human clinical trials. F[^18^F]FMISO-PET is noninvasive, clinically-available, and can provide information on temporal and spatial variations in hypoxia within tumors [37]. It has potential to open new opportunities for personalized and image-guided adaptive immunotherapy approaches.

## 5. Conclusions

Our mathematical model is able to capture a tumor’s response to evofosfamide and checkpoint blockade immunotherapy. The model also captures the interaction between these two types of treatments and their effects on a tumor’s vascularization. Notably, the model was able to capture the tumor’s temporal evolution observed in the five different treatment conditions (i.e., control, hypoxic and normoxic tumors treated with immunotherapy, treated with evofosfamide, and hypoxic tumors treated with immunotherapy and evofosfamide), with an average CCC of 0.86 ± 0.05. Interestingly, the model calibrated with parameter values from those five scenarios was not able to predict the response of normoxic tumors to combination immunotherapy and evofosfamide. However, increasing the tumor death rate due to immunotherapy (*γ*_*i*_) by a factor of 18.6 allowed us to predict the tumor growth with a CCC of 0.98. These results strongly indicate that evofosfamide not only increases the tumor vascularization, as considered in our model, but also increases efficacy of the immunotherapy, suggesting a synergistic interaction between the two treatments. This potential synergy warrants further investigation, and may have significant implications for the optimization of the dosing and scheduling of these drugs for maximal clinical benefit. This work provides a framework for identifying testable hypotheses for the effects and actions of evofosfamide and immunotherapy in the treatment of tumors.

## Supplementary Material

1

## Figures and Tables

**Table 1.** Parameter’s definitions and uniform priors. ***𝒰***(*a*, *b*) represents a uniform distribution with bounds between *a* and *b* for the prior values of the corresponding parameters.

Parameter	Meaning	Prior
*k*_*T*_	tumor growth rate	**𝒰**(0,0.4) *h*^−1^
*k*_*V*_	vascularization rate	**𝒰**(0,0.5) *h*^−1^
*μ*_*V*_	well-vascularized tumor fraction decay rate	**𝒰**(0,0.5) *h*^−1^
*μ*_*T*_	tumor death rate by immune system	**𝒰**(0,0.5) *h*^−1^
*γ*_*i*_	immunotherapy tumor death rate increase	**𝒰**(0,3.5) *h*^−1^
*r*_*i*_	immunotherapy effect decay rate	**𝒰**(0,0.2) *h*^−1^
*γ*_*e*_	evofosfamide vascularization rate increase	**𝒰**(0,3.5) *h*^−1^
*r*_*e*_	evofosfamide effect decay rate	**𝒰**(0,0.2) *h*^−1^

**Table 2.** Mean, and 16th and 84th percentiles (i.e., the standard deviation for a normal distribution) of the calibrated parameters.

Scenario	Parameter							
	*γ*_*e*_ (h^−1^)	*r*_*e*_ (h^−1^)	*γ*_*i*_ (h^−1^)	*k*_*T*_ (h^−1^)	*k*_*v*_ (h^−1^)	*μ*_*V*_ (h^−1^)	*μ*_*T*_ (h^−1^)	*r*_*i*_ (h^−1^)
I	n/a	n/a	0.022 [0.016, 0.029]	0.33 [0.31, 0.34]	0.094 [0.087, 0.10]	0.082 [0.071, 0.094]	0.36 [0.34, 0.39]	0.17 [0.14, 0.19]
II								
III								
IV	0.0091 [0.0052, 0.014]	0.14 [0.072,0.18]						
V								
VI			0.41 [0.37, 0.45]					
