# Supplementary material for: Predicting response to combination evofosfamide and immunotherapy under hypoxic conditions in murine models of colon cancer
Source: Math Biosci Eng. Author manuscript; Available in PMC 2023 Dec 7. (PMC10703000; doi:10.3934/mbe.2023783)
Supplement: 1 [file NIHMS1942375-supplement-1.pdf]

Supplementary

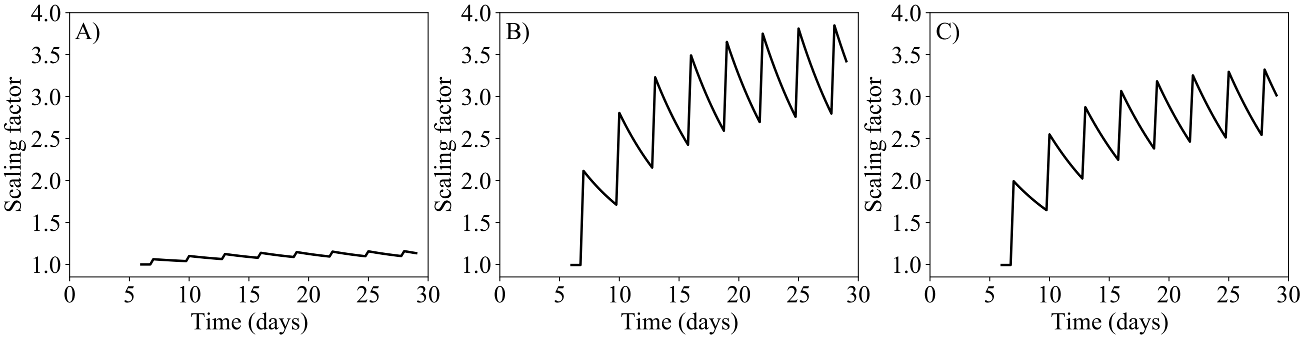

Figure S1. Scaling factor of the total tumor death rate (i.e., the tumor death rate by the immune system, constant in time, plus the increase in death rate by the immunotherapy, changing in time as the treatment is delivered) over time, computed as the ratio of the total tumor death rate in one treatment scenario to the total tumor death rate in another scenario. The scaling factor is shown for: A) immunotherapy compared to control, B) immunotherapy plus evofosfamide compared to control, and C), immunotherapy plus evofosfamide compared to immunotherapy. The average and standard deviation of the scaling factor in panels A, B, and C are 1.10 ± 0.04, 2.79 ± 0.66, and 2.52 ± 0.53, respectively. The scaling factor provides a useful way to visualize and compare the efficacy of different treatment scenarios in inducing tumor death.

AIMS Press

© 2023 the Author(s), licensee AIMS Press. This is an open access article distributed under the terms of the Creative Commons Attribution License (http://creativecommons.org/licenses/by/4.0)
